# *Helicobacter pylori* infection in children: an overview of global characteristics and the effectiveness of tailored therapy

**DOI:** 10.3389/fped.2026.1789563

**Published:** 2026-07-15

**Authors:** Marco Manfredi, Luca Braglia, Alessio Canovi, Giuseppe Pagliaro

**Affiliations:** 1 Pediatric Unit, Maternal and Child Department, Azienda USL-IRCCS di Reggio Emilia, Sant’Anna Hospital, Castelnovo ne’ Monti, Reggio Emilia, Italy; 2 Clinical Trials Center, Infrastruttura Ricerca e Statistica, Azienda USL-IRCCS di Reggio Emilia, Reggio Emilia, Italy; 3 Pediatric Gastroenterology Outpatient Clinic, Pediatric Unit, Maternal and Child Department, Azienda USL-IRCCS di Reggio Emilia, ASMN Hospital, Reggio Emilia, Italy

**Keywords:** antimicrobial susceptibility, children, clinical symptoms, endoscopic features, *Helicobacter pylori*

## Abstract

**Introduction:**

No specific symptomatology or typical endoscopic features exist for children infected with *Helicobacter pylori*, except for nodular gastritis, which, when present, is highly predictive of the infection. Despite international guidelines, empiric antibiotic eradication remains common. This study compares the clinical and endoscopic features of infected and non-infected children and evaluates antimicrobial resistance and eradication rates between tailored and empirical treatments.

**Methods:**

We retrospectively analyzed all children infected with *H. pylori* who were diagnosed by gastroscopy and histology over 3 years, matching them with *H. pylori*-negative controls. Infected children with known antibiotic susceptibility received tailored therapy, while others were treated empirically.

**Results:**

Clinical and endoscopic features did not differ significantly between the groups, confirming that one cannot diagnose *H. pylori* infection based on these features. Chronic active and non-active gastritis were present in all the infected and non-infected children, respectively. Clarithromycin and metronidazole resistance were 46.2% (6/13) and 23.1% (3/13), respectively. Eradication was obtained in 100% of the patients who received susceptibility-directed therapy compared to 75% in those who received empiric therapy.

**Discussion:**

We confirm that infected and non-infected children present with similar clinical and macroscopic features in gastroscopy and resistance to amoxicillin and tetracycline remains very low. While the small sample sizes limit sweeping conclusions, tailored therapy demonstrated maximum efficacy. Empiric regimens should be avoided if resistance testing is available. Alternatively, clinicians should select antibiotics with the lowest probability of resistance, such as amoxicillin, tetracycline, or metronidazole.

## Introduction

The global prevalence of *Helicobacter pylori* is approximately 50%, but infection rates vary between high-income and low-to-middle-income countries, with prevalences ranging between 20% and 80% (1). *H. pylori* infection is most frequently acquired during infancy or childhood, and close person-to-person contact is the primary transmission route, especially among those within the same household (2, 3).

The initial infection is generally asymptomatic, although children may present with nausea, vomiting, and abdominal or epigastric pain; the latter two symptoms frequently lead doctors to investigate for *H. pylori* infection in children (4, 5).

Several studies seem to support a causal relationship between *H. pylori* and various gastrointestinal and non-gastrointestinal diseases, such as iron deficiency anemia, idiopathic thrombocytopenic purpura (ITP), and failure to thrive (FTT) (5, 6). However, to date, causal relationships have yet to be confirmed with ITP and FTT. *H. pylori* clearly satisfies Koch's postulates as a causative agent for gastritis, duodenal and gastric ulcers, gastric adenocarcinoma, and gastric cancer. Numerous studies have confirmed that these gastroduodenal diseases are caused by, and not just associated with, *H. pylori* infection (5). For this reason, pediatric guidelines recommend a careful assessment of every infected child before deciding to perform gastroscopy and treat the infection, weighing the real expected benefits against the costs and risks of eradication treatment (4–8).

During gastroscopy, nodular antral mucosa is highly predictive of *H. pylori* infection, especially in pediatric patients, with a predictive value ranging between 46% and 73% (2). Beyond antral nodularity, during gastroscopy, infected children may have gastric superficial hyperemia, gastric erosions, and ulcers (9). Gastric erosions and ulcers only represent 1% of macroscopic diagnoses in children with *H. pylori* infection. However, the absence of antral nodularity does not preclude the diagnosis of *H. pylori* infection, which requires identification of the bacterium by histopathological exam (1, 2).

Antibiotic resistance has increased globally, making eradication of *H. pylori* more difficult, especially in pediatric patients, where the availability of antibiotics is lower than in adults (6, 10). Several previous studies in this field have shown that the clarithromycin resistance rate has increased in recent years and the metronidazole resistance rate has slowly decreased, though susceptibility remains under 80% (11). The metronidazole resistance can be overcome by increasing the dose to obtain higher success rates in empirical treatment, as recommended by the most recent European and North American Societies of Pediatric Gastroenterology, Hepatology and Nutrition (ESPGHAN/NASPGHAN) guidelines (4).

Culture by gastric biopsy is the most common and specific invasive diagnostic method used to detect the antimicrobial susceptibility of *H. pylori* in children, although its sensitivity is variable (60%–90%) (4), mainly due to several methodological factors, such as biopsy site, transport medium, time from sampling to processing, culture medium, and incubation conditions. One biopsy specimen from the antrum and one from the corpus should be taken separately to achieve optimal results (4, 12).

In children, the most recent ESPGHAN/NASPGHAN guidelines recommend antibiotic susceptibility testing for clarithromycin before starting clarithromycin-based triple therapy in areas/populations with a known high resistance rate (>20%) (4). In Italy, for example, the clarithromycin resistance rate is higher than 20% in both adults (13) and children (14). Once an *H. pylori* strain is resistant to clarithromycin, it is resistant to all macrolides, e.g., azithromycin (15).

The most common antibiotics used in *H. pylori* eradication treatment in children include amoxicillin, clarithromycin, and metronidazole. The use of quinolones in children is limited because juvenile animals developed arthropathy in previous experiments on fluoroquinolone use. However, these observations have not been clearly demonstrated in humans. The use of tetracycline is recommended in patients older than 8 years (10).

Pediatric guidelines recommend not treating without antimicrobial susceptibility testing unless this testing methodology is not available at the institution at which the child infected with *H. pylori* is being treated (4), in order to reduce antibiotic resistance and negative effects on intestinal microbiota.

The primary aim of the study is to verify the correlation between symptomatology and endoscopic features in infected and non-infected children. Second, we evaluated a snapshot of the antibiotic resistance rate in our geographical region and the differences in eradication between tailored and empirical treatments.

## Method

### Ethics statement

The study was conducted in accordance with the Declaration of Helsinki and approved by the Ethics Committee of AVEN, Italy (protocol code 783/2022/OSS/AUSLRE; date of approval 10 January 2023). Despite the retrospective nature of this study, we obtained written informed consent from parents or guardians.

We retrospectively reviewed all reports (33 patients) regarding children who arrived at our center from January 2019 to December 2021 because of the following symptoms: anemia, epigastric or abdominal pain, vomiting, and failure to thrive. We only enrolled the patients (18 children) with histologically confirmed *H. pylori* infection, since it is the gold standard for diagnosis (4). Twelve children with overlapping symptoms who had undergone gastroscopy were enrolled as the control group. The demographic and clinical characteristics of the patients are presented in the Supplementary Material. All the children underwent gastroscopy under anesthesia, with biopsies for histological examination and culture for *H. pylori*. In all the patients, at least two biopsies from the antrum and two from the corpus-fundus were taken, following the Updated Sydney Criteria (12). The endoscopic biopsy specimens were embedded in paraffin, sectioned, and stained with hematoxylin-eosin. Furthermore, one biopsy specimen from the antrum and one from the corpus-fundus were immediately placed in an appropriate transport medium (Portagerm-*Pylori*, bioMerieux, France) and then homogenized and cultured on both selective Pilory Agar (Biomerieux and Columbia Agar by Liofilchem, Italy) and non-selective (5% sheep blood agar) media. To determine susceptibility, we adopted the European Committee on Antimicrobial Susceptibility Testing (EUCAST) guidelines (http://www.eucast.org; accessed on 13 August 2024). The minimum inhibitory concentration (MIC) breakpoints for amoxicillin, clarithromycin, metronidazole, tetracycline, rifampicin, and levofloxacin are >0.125, >0.5, >8, >1, >1, and >1 mg/L, respectively. All the patients with known susceptibility received tailored therapy comprising amoxicillin + clarithromycin or metronidazole, based on their susceptibility, for 14 days. Children with double resistance to both clarithromycin and metronidazole received triple therapy with amoxicillin and metronidazole at a high dose for 14 days, as recommended by the guidelines (4). Finally, the children with unknown antimicrobial susceptibility were treated using an empirical regimen comprising amoxicillin and metronidazole at standard dosages for 14 days.

Regarding data analysis, after summarizing the sample’s main characteristics via descriptive statistics, diagnostic performance indexes for *H. pylori* presence were estimated for several symptoms (abdominal pain, short stature, vomiting, epigastric pain, and anemia), starting from 2  ×  2 tables, and were accompanied by 95% confidence intervals estimated using the Clopper–Pearson method. Table 1 shows the confusion matrix for the symptoms, while the diagnostic performances are shown in the Supplementary Materials.

**Table 1:** Symptom confusion matrix. A confusion matrix is a cross-classification table indicating diagnostic performance, with the gold/reference standard in the columns, diagnostic tests in the rows, and frequency in the cells. Here, several are reported and stacked, with each one considering a single sign/symptom in turn, in order to provide a full descriptive account of the sample.

Symptom presentation	*H. pylori*-POS	*H. pylori*-NEG	Total	Estimate	Value (95% CI)
With abdominal pain	2	2	4	Sensitivity	11.1% (1.4%–34.7%)
Without	16	10	26	Specificity	83.3% (51.6%–97.9%)
Total	18	12	30	Accuracy	40.0% (22.7%–59.4%)
				PPV	50.0% (6.8%–93.2%)
				NPV	38.5% (20.2%–59.4%)
With failure to thrive	4	3	7	Sensitivity	22.2% (6.4%–47.6%)
Without	14	9	23	Specificity	75.0% (42.8%–94.5%)
Total	18	12	30	Accuracy	43.3% (25.5%–62.6%)
				PPV	57.1% (18.4%–90.1%)
				NPV	39.1% (19.7%–61.5%)
Vomiting	6	3	9	Sensitivity	33.3% (13.3%–59.0%)
No vomiting	12	9	21	Specificity	75.0% (42.8%–94.5%)
Total	18	12	30	Accuracy	50.0% (31.3%–68.7%)
				PPV	66.7% (29.9%–92.5%)
				NPV	42.9% (21.8%–66.0%)
With epigastric pain	10	10	20	Sensitivity	55.6% (30.8%–78.5%)
Without	8	2	10	Specificity	16.7% (2.1%–48.4%)
Total	18	12	30	Accuracy	40.0% (22.7%–59.4%)
				PPV	50.0% (27.2%–72.8%)
				NPV	20.0% (2.5%–55.6%)
With anemia	4	0	4	Sensitivity	22.2% (6.4%–47.6%)
Without	14	12	26	Specificity	100.0% (73.5%–100.0%)
Total	18	12	30	Accuracy	53.3% (34.3%–71.7%)
				PPV	100.0% (39.8%–100.0%)
				NPV	46.2% (26.6%–66.6%)
With SAT	15	0	15	Sensitivity	100.0% (78.2%–100.0%)
Without	0	8	8	Specificity	100.0% (63.1%–100.0%)
Total	15	8	23	Accuracy	100.0% (85.2%–100.0%)
				PPV	100.0% (78.2%–100.0%)
				NPV	100.0% (63.1%–100.0%)

Regarding secondary analyses, the associations between endoscopic/histologic variables and diagnostic tests were explored univariately using cross-tabulations and Fisher’s exact test; antibiotic resistance (several drugs were evaluated for all patients) was estimated as a percentage and compared using Cochran’s Q test for paired proportions. Finally, the association between the antibiotic scheme and eradication was explored using cross-tabulations and Fisher’s exact test.

Statistical analysis was performed using R version 4.3.0.

For each group, we analyzed symptoms and endoscopic and histological features.

## Results

### General cohort characteristics

Of the 33 patients who fulfilled the inclusion criteria, 30 had undergone gastroscopy and were included in the study, with 18 children testing positive (Group A) and 12 negative (Group B) (prevalence of 60%, 95% CI: 40.6% to 77.3%).

### Comparison of symptoms/endoscopic findings between the two groups

As reported above, the symptoms of enrolled patients who had undergone gastroscopy were anemia, epigastric or abdominal pain, vomiting, and failure to thrive. We did not find differences regarding symptomatology between the infected and non-infected children. The 2 × 2 cross-tabulations of symptoms with diagnostic performance are presented in Table 1; in all cases but one, the marker appeared to show either high sensitivity or high specificity. The best results were obtained with stool antigen, which perfectly predicted the presence of *H. pylori*.

As a primary analysis, we compared the symptomatology and endoscopic characteristics of the infected and non-infected children. These results showed no differences between the two groups (Tables 2a,b).

**Table 2:** Comparison of endoscopic characteristics (a) and symptomatology between infected and non-infected children (b).

(a) Comparison of endoscopic features between *H. pylori*-positive and *H. pylori*-negative symptomatic children			
Endoscopic feature	*H. pylori*-positive (*N* = 18)	*H. pylori*-negative (*N* = 12)	*P*-value*
Antral nodularity, *n* (%)	3 (16.7%)	0 (0.0%)	0.255
Antral hyperemia, *n* (%)	15 (83.3%)	10 (83.3%)	1.000
Normal mucosa, *n* (%)	3 (16.7%)	2 (16.7%)	1.000

(b) Comparison of clinical symptoms between *H. pylori*-positive and *H. pylori*-negative symptomatic children			
Symptom	*H. pylori*-positive (*N* = 18)	*H. pylori-*negative (*N* = 12)	*P*-value*
Epigastric pain, *n* (%)	11 (61.1%)	10 (83.3%)	0.443
Abdominal pain, *n* (%)	3 (16.7%)	2 (16.7%)	1.000
Failure to thrive, *n* (%)	4 (22.2%)	3 (25.0%)	1.000
Vomiting, *n* (%)	5 (27.8%)	3 (25.0%)	1.000
Anemia, *n* (%)	4 (22.2%)	0 (0.0%)	0.266

The three patients with nodular features also had antral mucosal hyperemia.

The overall cumulative statistical distribution in the two groups showed no significant difference (global Fisher's exact test for the 3 × 2 table, *P* = 0.441).

* *P*-values for individual rows were calculated using a 2 × 2 Fisher's exact test (presence vs. absence of each feature).

The cumulative distribution of symptoms in the two groups showed no global statistically significant difference (global Fisher's exact test for the 5 × 2 table, *P* = 0.686).

* *P*-values were calculated using a 2 × 2 Fisher's exact test (presence vs. absence of each individual symptom).

As a secondary analysis, we analyzed the associations between endoscopic/histologic variables and signs/symptoms. We opted for univariate analyses given the data scarcity/skewness. Each factor was investigated using cross-tabulation and association analysis. Statistically significant associations were found between the following:

- anemia and nodular aspects of gastric mucosa (*p* = 0.039);
- type of gastritis and anemia (*p* = 0.023); and
- type of gastritis and stool antigen (*p* < 0.001).

These data are presented in the Supplementary Material.

The main symptoms of the 18 infected children were epigastric pain (11 children, 52.3%), vomiting (6 children, 28.6%), and failure to thrive (5 children, 23.8%), the latter defined as a weight-for-age Z-score less than −2 on standardized growth charts for age and sex according to WHO guidelines (https://www.who.int/tools/child-growth-standards/standards). Several patients had more than one symptom simultaneously.

During endoscopy, a macroscopic nodular appearance of the gastric mucosa was reported in 3 out of 18 *H. pylori*-positive children and in none of the non-infected patients (*p* = 0.255). Antral hyperemia was present in 15 infected children and 10 non-infected controls, respectively (*p* = 1.000). All 18 infected children had chronic active gastritis at histological examination. Only one child had a peptic ulcer, and his symptomatology was restricted to vomiting. In the control group, all the children had chronic non-active gastritis.

### Antimicrobial resistance rates

Culture was performed in only 13/18 patients, and in all of these, antimicrobial susceptibility testing was performed.

Resistance percentages (with confidence intervals) are presented in Table 3, with Cochran’s Q test demonstrating statistically significant differences between them (*p* = 0.005). All the infected patients were susceptible to amoxicillin. The patients with antimicrobial susceptibility received amoxicillin and either clarithromycin or metronidazole, based on their susceptibility, for 14 days. The patients with unknown antimicrobial susceptibility received amoxicillin and metronidazole at standard dosages for 14 days. Beyond antibiotics, all patients received a proton pump inhibitor. All drugs were administered twice a day.

**Table 3:** Antibiotic resistance rates among 13/18 infected patients.

Antibiotic	MIC (mg/mL)	*N*	Resistant	Resistance (95% CI)
Clarithromycin	>0.5	13	6	46.2% (19.2%–74.9%)
Metronidazole	>8	13	3	23.1% (5.0%–53.8%)
Levofloxacin	>1	13	3	23.1% (5.0%–53.8%)
Amoxicillin	>0.125	13	0	0% (0%–24.7%)
Tetracycline	>1	13	0	0% (0%–24.7%)

Descriptively speaking (given the width of the coefficient intervals due to the small sample size), in our sample, clarithromycin and metronidazole resistance rates were high; furthermore, the levofloxacin resistance rate was higher than 20%, but quinolones are not recommended in children (9). Overall, resistance to different antibiotics was found to be significantly different (Cochran’s Q test *p* = 0.005).

### Treatment efficacy

Only 13/18 patients underwent the infection eradication tes; 9 out of 13 and 4 out of 5 patients had known and unknown antimicrobial susceptibility, respectively.

All the children with full susceptibility had successfully eradicated infections, whereas only one child out of the four treated empirically had eradicated infections (Table 4).

**Table 4:** Eradication of infection among the empirically treated children (Fisher’s exact test *p* = 1.000).

Treatment efficacy	CLA + MET	%	CLA	%	MET	%	N	%	Total	%
Not eradicated	0	0.00	2	50.00	1	25.00	0		3	33.33
Eradicated	1	100.00	2	50.00	3	75.00	0		6	66.67
Missing	1		5		1		14		21	
Total	2		9		5		14		30	

One out of the two children with double resistance to both clarithromycin and metronidazole who were treated with amoxicillin + metronidazole at a high dose for 14 days had an eradicated infection. The mother of the child who did not have an eradicated infection reported that she only administered the therapy for 4 days and did not finish the treatment.

Cross-tabulations of these data are presented in Table 4 (no statistical differences were found).

Five patients out of 18 (four with known and one with unknown antimicrobial susceptibility, respectively) did not undergo an eradication test after therapy, and therefore, they were excluded from the analysis.

## Discussion

*H. pylori* infection is one of the most widespread infections in humans. Although it is acquired during childhood, infected children do not develop significant symptoms compared to adults. It is still quite controversial whether *H. pylori* plays a protective role in children against inflammatory bowel disease (IBD), asthma, allergy, and gastroesophageal reflux disease (GERD) (5). For these reasons, pediatric international guidelines recommend fully evaluating all possible causes before diagnosing and treating the infection (4). In contrast, the recent American College of Gastroenterology (ACG) clinical guidelines recommend proactive examinations of infected adults and all positive patients should receive effective eradication therapy (16).

Our results, besides confirming the varied spectrum of symptoms in children infected with *H. pylori* (17), with no significant difference between the infected and non-infected groups, also provide additional evidence to support the ongoing clinical challenge faced in pediatric gastroenterology with respect to the lack of relationships between reportable symptoms and mucosal disease severity. With respect to the nodular antral mucosa in infected patients, there is consensus in the literature that antral nodularity is a predictor of *H. pylori* infection in children (17). In our study, macroscopic antral nodularity was uniquely found in the *H. pylori*-positive group, confirming its high diagnostic specificity for the infection. Conversely, no difference was noted between the two groups regarding the prevalence of antral mucosal hyperemia. Moreover, all the infected children had chronic active gastritis and stool antigen tests (SAT) perfectly predicted the presence of *H. pylori*.

Recently, the prevalence of antibiotic-resistant *H. pylori* has steadily increased globally, mainly due to the inappropriate use of antibiotics in both adults and children. The World Health Organization has recently included clarithromycin-resistant *H. pylori* strains in the priority pathogens list for the research and development of new antibiotics (18). Nonetheless, in our geographic region, many infected children, unfortunately, are often treated empirically, despite tailored therapy providing a better eradication rate (19). In addition, many doctors frequently do not perform eradication tests after treatment. These are not only essential to know whether the infection has been eradicated, but also crucial in understanding the possible onset of antibiotic resistance. Culturing of *H. pylori*, through gastroscopy, is thus far the only method for determining antibiotic susceptibility in children, at least in Italy, but it is an invasive method. Furthermore, it is limited by several factors, such as the suitability of gastric specimens (we require at least one specimen from the antrum and one from the gastric body to increase the chance of development); an appropriate method of preservation, mainly during transport to the laboratory; and the intrinsic difficulty of culturing *H. pylori* (4, 12). In our study, cultures were developed for all patients. We believe we obtained a very high development rate because the endoscopic rooms are very close to the microbiology laboratory, thus greatly reducing the interval between sampling and laboratory processing, a key step for assuring a higher rate of susceptibility development (20).

Amoxicillin is the main key factor in the eradication of *H. pylori*. In our area, the amoxicillin resistance rate has always been very low (11, 14, 21, 22). In contrast, clarithromycin resistance is the main cause of unsuccessful eradication worldwide because of its widespread and often indiscriminate use (4). Our study confirms high resistance rates for both clarithromycin and metronidazole, both of which were higher than 20%.

In Europe, the amoxicillin resistance rate is very low, except in Germany (approximately 20%) (23). However, in a Belgian study, the clarithromycin resistance rate was found to be very high (> 20%) in Europe (24, 25). The metronidazole resistance rate is particularly higher in developing countries because of its use for parasitic infections (26). Our study confirms that resistance to tetracycline, like amoxicillin, is very rare in children (11, 14, 21, 22, 24, 25), but tetracycline is suitable only in children older than 8 years (4, 10, 27). In addition, the ciprofloxacin resistance rate remains >20%, although quinolones are not usually recommended in pediatric patients (10). Recent studies analyzed the antibiotic resistance rates in both *H. pylori*-infected adults and children in Italy and in our area. The clarithromycin and metronidazole resistance rates were between 30% and 40% in adults (21, 28, 29). Among children in our area, the clarithromycin and metronidazole resistance rates were both approximately 25% (14).

Despite the low number of patients, this study clearly showed that antibiotic susceptibility-tailored therapy is far more effective in achieving eradication success than empiric treatment (100% vs. 75%, respectively), strengthening the recommendations of guidelines to this end. The metronidazole resistance rate was slightly lower, while the clarithromycin resistance rate was higher than 15%, compared to equivalent studies conducted in 2015 and 2018 (11, 14). The decline in sensitivity to clarithromycin is probably due to its widespread and often indiscriminate use for common pediatric airway infections.

Since the amoxicillin and tetracycline resistance rates were confirmed to be very low, these antibiotics (amoxicillin, tetracycline, and metronidazole) are better choices for empiric therapy.

Due to the increasing antibiotic resistance rate, treatment failures in patients infected with *H. pylori* have become a major problem worldwide. Unfortunately, the availability of antibiotics in children is lower compared to adults; therefore, it is always more important to treat after individual susceptibility testing, so as not to waste antibiotics. Regarding the two patients with double resistance to clarithromycin and metronidazole, only one had an eradicated infection through triple therapy with a high dose of metronidazole, as recommended by the guidelines (4). The other patient did not correctly complete the therapeutic regimen.

Hence, in pediatrics, because of the limited availability of antibiotics, the problem of how to treat patients with double resistance (clarithromycin and metronidazole) remains an important clinical and public health problem that is not easily resolvable in the short term. In our series, as the most recent ESPGHAN/NASPGHAN guidelines recommend, triple therapy comprising amoxicillin + metronidazole at a high dosage appears to be the most effective. International pediatric guidelines recommend not using clarithromycin in empirical regimens (4). This recommendation is confirmed by our study.

Our study has some limitations. First, there was a small sample of patients, which is due to this data being from the COVID-19 pandemic, when people hesitated to go to the doctor. However, our results confirm those of previous studies with larger samples. We decided to perform this retrospective study in 2022 and analyze the previous three years. When we realized there would be a small number of patients, the local Ethics Committee had already approved the study, and it would have been problematic to submit it again to the Committee. Thus, further studies with larger samples are needed to confirm or deny our results.

Second, some of the patients were withdrawn at follow-up because conducting an eradication test was a general practitioner's responsibility, resulting in a reduced eradication rate. In this regard, we are taking on this task in order to regularly update the general practitioners of our district.

In addition, there was a high rate of follow-up loss (four patients in each group, thus 38% of total patients did not undergo the post-treatment testing and were excluded from analysis. Therefore, the analyzed sample was further reduced, affecting the robustness of the efficacy conclusions.

In conclusion, considering the variable symptomatology of *H. pylori* infection in children, it is not possible to assume that the infection is present based on reported clinical symptoms alone or even through macroscopic features on gastroscopy. Because of the stable increased antibiotic resistance rates and limited antibiotic availability in pediatrics, we should always attempt to treat children infected with *H. pylori* after individual antimicrobial susceptibility testing. Therefore, empiric treatment for *H. pylori* should only be administered using the antibiotics (e.g., amoxicillin and metronidazole) that the infecting strain is least likely be resistant to. At the same time, these antibiotics, following the ESPGHAN/NASPGHAN recommendations, should be used in countries where antimicrobial susceptibility testing is not available.

## Data Availability

The raw data supporting the conclusions of this article will be made available by the authors, without undue reservation.
