# Supplementary material for: Helicobacter pylori infection in children: an overview of global characteristics and the effectiveness of tailored therapy
Source: Front Pediatr. 2026 Jul 15;14:1789563. doi: 10.3389/fped.2026.1789563 (PMC13417099; doi:10.3389/fped.2026.1789563)
Supplement: Supplementary file 1 [file Supplementaryfile1.docx]

Tables as supplementary files

**Table 1.** Demographics of the sample.

| Overall | Variables |  |
| --- | --- | --- |
| 30 | n |  |
| 12 (40.0) | Sez= F (%) |  |
| 9.95 [6.90, 12.38] | age (median [IQR]) |  |
|  | Ethnicity (%) |  |
| 22 (73.4) | Western Europe |  |
| 1 (3.33) | Eastern Europe |  |
| 4 (13.3) | North African |  |
| 3 (10.0) | Subsaharian |  |

Table 2: Association between endoscopic/histological characteristics and tests

| **Endoscopic/histological** | **Tests** | **p** |
| --- | --- | --- |
| Nodular gastritis | Abdominal pain | 0.360 |
| Nodular gastritis | Failure to thrive | 1.000 |
| Nodular gastritis | Vomiting | 1.000 |
| Nodular gastritis | Epigastric pain | 0.251 |
| Nodular gastritis | Anemia | 0.039 |
| Nodular gastritis | SAT | 0.526 |
| Type of gastritis | Abdominal pain | 1.000 |
| Type of gastritis | Failure to thrive | 0.783 |
| Type of gastritis | Vomiting | 0.650 |
| Type of gastritis | Epigastric pain | 0.151 |
| Type of gastritis | Anemia | 0.023 |
| Type of gastritis | SAT | <0.001 |
| Peptic ulcer | Abdominal pain | 1.000 |
| Peptic ulcer | Failure to thrive | 1.000 |
| Peptic ulcer | Vomiting | 0.300 |
| Peptic ulcer | Epigastric pain | 0.333 |
| Peptic ulcer | Anemia | 1.000 |
| Peptic ulcer | SAT | 1.000 |

Table 3. Clinical symptomatology of the infected children.

| **Symptomatology** | **Freq (n = 18)** | **%** |
| --- | --- | --- |
| Epigastric pain | 11 | 61.1 |
| Vomiting | 6 | 33.3 |
| Failure to thrive | 5 | 27.8 |
| Iron deficiency anemia | 4 | 22.2 |
| Abdominal pain | 2 | 11.1 |

* Some children had more than one symptom, simultaneously.

Table 4: Diagnostic performance of abdominal pain

|  | **Estimate** | **Lower 95% CI** | **Upper 95% CI** |
| --- | --- | --- | --- |
| Sensitivity | 0.111 | 0.014 | 0.347 |
| Specificity | 0.833 | 0.516 | 0.979 |
| Accuracy | 0.400 | 0.227 | 0.594 |
| PPV | 0.500 | 0.068 | 0.932 |
| NPV | 0.385 | 0.202 | 0.594 |

Table 5: Diagnostic performance of failure to thrive

|  | **Estimate** | **Lower 95% CI** | **Upper 95% CI** |
| --- | --- | --- | --- |
| Sensitivity | 0.222 | 0.064 | 0.476 |
| Specificity | 0.750 | 0.428 | 0.945 |
| Accuracy | 0.433 | 0.255 | 0.626 |
| PPV | 0.571 | 0.184 | 0.901 |
| NPV | 0.391 | 0.197 | 0.615 |

Table 6: Diagnostic performance of vomiting

|  | **Estimate** | **Lower 95% CI** | **Upper 95% CI** |
| --- | --- | --- | --- |
| Sensitivity | 0.333 | 0.133 | 0.590 |
| Specificity | 0.750 | 0.428 | 0.945 |
| Accuracy | 0.500 | 0.313 | 0.687 |
| PPV | 0.667 | 0.299 | 0.925 |
| NPV | 0.429 | 0.218 | 0.660 |

Table 7: Diagnostic performance of epigastric pain

|  | **Estimate** | **Lower 95% CI** | **Upper 95% CI** |
| --- | --- | --- | --- |
| Sensitivity | 0.556 | 0.308 | 0.785 |
| Specificity | 0.167 | 0.021 | 0.484 |
| Accuracy | 0.400 | 0.227 | 0.594 |
| PPV | 0.500 | 0.272 | 0.728 |
| NPV | 0.200 | 0.025 | 0.556 |

Table 8: Diagnostic performance of anemia

|  | **Estimate** | **Lower 95% CI** | **Upper 95% CI** |
| --- | --- | --- | --- |
| Sensitivity | 0.222 | 0.064 | 0.476 |
| Specificity | 1.000 | 0.735 | 1.000 |
| Accuracy | 0.533 | 0.343 | 0.717 |
| PPV | 1.000 | 0.398 | 1.000 |
| NPV | 0.462 | 0.266 | 0.666 |

Table 9: Diagnostic performance values of SAT

|  | **Estimate** | **Lower 95% CI** | **Upper 95% CI** |
| --- | --- | --- | --- |
| Sensitivity | 1.000 | 0.782 | 1.000 |
| Specificity | 1.000 | 0.631 | 1.000 |
| Accuracy | 1.000 | 0.852 | 1.000 |
| PPV | 1.000 | 0.782 | 1.000 |
| NPV | 1.000 | 0.631 | 1.000 |

Table 10: Correlation Between Stool Antigen Test Results and Histological Gastritis Activity

| **Stool Antigen Test** | **Active Chronic Gastritis  *n (%)*** | **Inactive Chronic Gastritis  *n (%)*** | **P-value *** |
| --- | --- | --- | --- |
| **Positive** *(N = 18)* | 18 (100.0%) | 0 (0.0%) | **< 0.001** |
| **Negative** *(N = 12)* | 0 (0.0%) | 12 (100.0%) |  |

** P-value was calculated using a 2×2 Fisher's exact test.*

Table 11. Correlation Between the Presence of Anemia and Histological Gastritis Activity

| **Anemia** | **Active Chronic Gastritis  *n (%)*** | **Inactive Chronic Gastritis  *n (%)*** | **P-value *** |
| --- | --- | --- | --- |
| **Yes** *(N = 4)* | 4 (100.0%) | 0 (0.0%) | **0.002** |
| **No** *(N = 26)* | 8 (30.8%) | 18 (69.2%) |  |

** P-value was calculated using a 2×2 Fisher's exact test.*

|  |  |  |  |  |  |  |  |  |  |  |  |
| --- | --- | --- | --- | --- | --- | --- | --- | --- | --- | --- | --- |
